# Myotropic Activities of Tick Pyrokinin Neuropeptides and Analog in Feeding Tissues of Hard Ticks (Ixodidae)

**DOI:** 10.3389/fphys.2021.826399

**Published:** 2022-02-15

**Authors:** Caixing Xiong, Juan P. Wulff, Ronald J. Nachman, Patricia V. Pietrantonio

**Affiliations:** ^1^Department of Entomology, Texas A&M University, College Station, TX, United States; ^2^Insect Neuropeptide Lab, Insect Control and Cotton Disease Research Unit, Southern Plains Agricultural Research Center, USDA-ARS, College Station, TX, United States

**Keywords:** acari, muscle contraction, mouthpart, PK/PBAN, vector biology, tick physiology, feeding disruption, PVK

## Abstract

Neuropeptides regulate many important physiological processes in animals. The G protein-coupled receptors of corresponding small neuropeptide ligands are considered promising targets for controlling arthropod pests. Pyrokinins (PKs) are pleiotropic neuropeptides that, in some insect species, stimulate muscle contraction and modulate pheromone biosynthesis, embryonic diapause, and feeding behavior. However, their function remains unknown in ticks. In this study, we reported the myotropic activity of tick endogenous PKs and a PK agonist analog, PK-PEG_8_ (MS[PEG_8_]-YFTPRLa), on feeding tissues of two tick species representing the family Ixodidae lineages, namely, Prostriata (*Ixodes scapularis*) and Metastriata (*Rhipicephalus sanguineus*). First, we predicted the sequences of two periviscerokinins (PVK), one with a derived ending RNa and five PKs encoded by the CAPA peptide precursor from *R. sanguineus* and found the encoded PKs were identical to those of *R. microplus* identified previously. The pharynx-esophagus of both tick species responded with increased contractions to 10 μM of the endogenous PK as well as to PK-PEG_8_ but not to the scrambled PK peptide, as expected. A dose-dependent myotropic activity of the PK-PEG_8_ was found for both tick species, validating the analog activity previously found in the pyrokinin recombinant receptor assay. In agreement with the tissue activity elicited, we quantified the relative transcript abundance of *R. sanguineus* PK receptor in unfed female ticks and found it was the highest in the feeding tissues extracted from the capitulum and lowest in the reproductive tissue. This is the first report of the activity of pyrokinins in ticks. These findings strongly indicate the potential role of PKs in regulating tick blood feeding and therefore, making the tick PK receptor a potential target for interference.

## Introduction

G protein-coupled receptors (GPCRs) are promising novel targets for tick control (Pietrantonio et al., 2018). The success of amitraz as an acaricide that targets the tick octopamine/tyramine receptor is a proof-of-principle (Kita et al., 2017). Efforts have been made in academic settings toward discovering new chemistries against tick GPCR targets such as the leucokinin and dopamine receptors (Hill et al., 2013; Xiong et al., 2021a). In contrast to the rapid advance in the identification of ligands and GPCRs by means of omics tools, studies on the physiological function of tick neuropeptides are still limited. Such knowledge is essential for selecting GPCRs for screening synthetic chemical libraries and for understanding the mode of action of novel chemistries.

The invertebrate pyrokinin signaling system is putatively homologous to the neuromedin U system in vertebrates (Park et al., 2002). The CAPA/pyrokinin (PK) neuropeptide signaling system arose early in evolution as it is present in the nematode, *Caenorhabditis elegans* (Lindemans et al., 2009). The ancestral *capa/pk* gene encoding PKs was duplicated and differentiated in insects into two genes, namely, *capa* and *pk* (Diesner et al., 2021); the duplication of *capa/pk* gene probably occurred in the last common ancestor of Hexapoda and Remipedia (Derst et al., 2016). The pyrokinin was first discovered from the Madeira cockroach, *Leucophaea maderae*, as it elicited the myotropic activity of the hindgut (Holman et al., 1986). PKs from the American cockroach, *Periplaneta americana*, show myostimulatory activity on several tissues such as the hyperneural muscle, hindgut, foregut, and oviducts (Predel and Nachman, 2001), and pyrokinins also increase the contraction in the heart muscle of *Drosophila* (Meng et al., 2002). Later, PKs were found to be pleiotropic neuropeptides, which modulate physiological processes such as stimulation of sex pheromone biosynthesis in moths (Raina et al., 1989), melanization of cuticle in Lepidopteran larvae (Matsumoto et al., 1990), induction of embryonic diapause in *Bombyx mori* (Imai et al., 1991), and termination of pupal diapause in heliothine moths (Xu and Denlinger, 2003; Zhang et al., 2011). PKs accelerate pupariation in the flesh fly (Nachman et al., 2006).

Insect PKs feature a common C-terminal FXPRLamide sequence (Holman et al., 1986; Jurenka, 2015), and this amidated peptide fragment is the minimal required for biological activity (Nachman et al., 1986; Kim et al., 2008). The pyrokinin receptors from the ticks *Rhipicephalus microplus* and *Ixodes scapularis* were previously deorphanized (Yang et al., 2015; Gondalia et al., 2016), the corresponding CAPA propeptide transcripts were cloned, and the functional activity of putative endogenous ligands on the recombinant receptors was validated (Gondalia et al., 2016; Xiong et al., 2021b). The recombinant tick PK receptor is less ligand-selective for activation, as replacement of the last amino acid leucine with valine or isoleucine in the minimal active core does not change the ligand-receptor activity (Yang et al., 2015). The Ixsc-CAPA-PKs detected by mass spectrometry were all encoded by a single *capa* gene, and it appears that there is only one gene encoding the pyrokinin receptor in ticks (Yang et al., 2015; Gondalia et al., 2016). The tick PK signaling system appears to be ancestral to the insect taxa because the latter has gene duplications in both ligand and receptor genes (Jurenka and Nusawardani, 2011). However, the exact physiological function of tick pyrokinin remains unknown. In this study, we filled this knowledge gap by revealing the myotropic activity of tick pyrokinin and a PK analog in tick feeding tissues on two tick vector species, namely, the brown dog tick, *R. sanguineus*, and the blacklegged tick, *Ix. scapularis*.

## Materials and Methods

### Curation of *capa* Gene From *Rhipicephalus sanguineus*

To identify the *capa* gene from *R. sanguineus*, a BLASTn search was performed on NCBI against the *R. sanguineus* nucleotide collection (nr/nt) using the *capa* cDNA sequence we previously cloned from *R. microplus* (GenBank accession number MZ686950) (Xiong et al., 2021b). The identified *R. sanguineus* sequence was analyzed as follows: The signal peptide of the translated *capa* gene precursor sequence was predicted using SignaIP version 5.0 (Armenteros et al., 2019). The cleavage sites on the precursor were predicted following the principles summarized by Veenstra (2000).

### Ticks and Reagents

Unfed adult female *R. sanguineus* ticks were obtained from Ecto Services, Inc (Henderson, NC, United States). Unfed adult female *Ix. scapularis* ticks were obtained from Tick Rearing Facility at Oklahoma State University, United States. Tick colonies were maintained at 26.5 ± 1°C, in a 16:8 h (light: dark) photoperiod and under 97% relative humidity that was maintained by using a saturated potassium sulfate solution (Thangamani and Bente, 2014). *R. sanguineus* ticks were 2–3 months old, and *Ix. scapularis* ticks were 3–4 months old. Four peptides were used in this study, namely, Rhisa-CAPA-PK1 (RSNTFTPRIa), Ixosc-CAPA-PK1 (RSNNFTPRIa), a PK analog (PEG-P_8_: MS[PEG_8_]-YFTPRLa) designed based on insect PKs (Nachman et al., 2012), and a scrambled peptide (RNFSRINTPa) of Ixosc-CAPA-PK1 as negative control. All peptides were synthesized by GenScript^®^ Biotech (Piscataway, NJ, United States), and the peptide analog was synthesized as described by Nachman et al. (2012). The peptides were solubilized and diluted in a tick physiological saline solution, as described by Šimo et al. (2014), consisting of 140 mM NaCl (Macron, Central Valley, PA, United States, 5 mM KCl (Millipore-Sigma, St. Louis, MO, United States), 1 mM MgCl_2_ (Millipore-Sigma, St. Louis, MO, United States), 5 mM CaCl_2_ (EM Science, Gibbstown, NJ, United States), 4 mM NaHCO_3_ (EMD Chemicals, Gibbstown, NJ, United States), and 5 mM HEPES (Millipore-Sigma, St. Louis, MO, United States) at pH 7.2. Peptides and saline were kept on ice and transferred to a heat block (VWR Scientific Inc., Wayne, PA, United States), set at 26 ± 1°C, ∼30 min before the start of the assay.

### Tick Preparation for Contraction Assay

Individual ticks were immobilized by submerging their legs on a melted wax plate and dissected under the ice-cold tick physiological saline using a stereo microscope (Olympus, Center Valley, PA, United States) following the dissection protocol described by Tidwell et al. (2021). The ticks were incised along the dorsal alloscutum using a surgical blade (#12, Miltex^®^ Instruments, Princeton, NJ, United States). To expose the pharynx-esophagus, other tissues including midgut, trachea, rectal sac, reproductive tissue (ReprT), and synganglion (SynG) were removed using fine forceps. After dissection, ticks were transferred to a Sylgard^®^ (Dow Inc., Midland, MI, United States) filled Petri dish plate of 6 cm in diameter (Corning Inc., Corning, NY, United States) with fresh saline (100 μl), which can fully cover the tick, and the tick was pinned on the carcass using a #15 Minuten pin (BioQuip^®^ Product Inc., Rancho Dominguez, CA, United States). The peristaltic movement of the pharynx-esophagus was observed. Saline and peptide solution used for the contraction assay were kept at 26 ± 1°C before being applied on the tick tissue. The Sylgard^®^ plate was cleaned with 70% ethanol, 0.1 N NaOH, 0.1 N HCl, and deionized water each for three times between each experiment (tick) to completely remove any residual peptides.

### Determination of Activities of Pyrokinin and Pyrokinin Analog

All tissue responses were filmed for 1 min at 3 min after any treatment (saline, PK peptide, or analog) using a Lumenera Infinity-1 color camera (Teledyne Lumenera, Ottawa, ON, Canada) installed on an Olympus SZ61 trinocular stereo microscope (Olympus, Center Valley, PA, United States). Three treatments were performed on each tick in the following order: first, application of fresh saline, second, application of the scrambled peptide, and finally either the PK peptide or analog. First, the tick tissue was allowed to be stabilized for 5 min in the saline at RT, and then this first saline was replaced with fresh saline at 26 ± 1°C to register any background contractions. The saline was replaced by 100 μl of 10 μM scrambled peptide at 26 ± 1°C as a negative control and incubated for 3 min before the tissue response was filmed. Tissue was then rinsed five times within 1 min with 100 μl of saline, and this was replaced with 100 μl of 10 μM tick endogenous pyrokinin (Rhisa-CAPA-PK1 or Ixosc-CAPA-PK1) or the PK analog (PK-PEG_8_) at 26 ± 1°C. After 3 min of incubation, the tissue was filmed. Contractions of the pharynx-esophagus were counted by the same operator. Each peptide was tested on 6–7 ticks.

### Dose-Response Contraction Assay

To test the dose dependency of the contraction of *R. sanguineus* pharynx-esophagus in response to the endogenous pyrokinin and/or PK analog, the pharynx-esophagus tissue was prepared as described in the section “Tick Preparation for Contraction Assay.” Rhisa-CAPA-PK1 and PK-PEG_8_ were tested at five concentrations, namely, 0.1, 0.3, 1, 3, and 10 μM. The tissue responses (from the same tick) to the saline and the PK peptide or analog (from lowest to highest concentration) were recorded for a continuous 1 min after a short incubation (1 min) with each treatment. The tissue was rinsed five times with 100 μl saline before being treated with a higher peptide concentration. Contractions of pharynx-esophagus were counted by the same operator (*N* = 6–9 ticks).

### Verification of Expression of Pyrokinin Receptor Transcript in *R. sanguineus*

#### Tissue Collection, RNA Extraction, and cDNA Synthesis

Unfed 3-month-old *R. sanguineus* female ticks (Ecto Services, Inc., Henderson, NC, United States) were used for gene expression analyses. Ticks were dissected under physiological tick saline as described above and multiple tissues including ReprTs, SynG, pharynx-esophagus, chelicerate, and other soft tissues associated with the capitulum (PECO) and the rest of the body (ReB) were kept at –80°C until RNA isolation stored in 150 μl (SynG and PECO) or 250 μl (ReprT and ReB) of TRIzol™ reagent (Invitrogen; Carlsbad, CA, United States). ReprT consisted of all the soft tissue pulled from the gonopore, including vagina, seminal receptacle, oviducts, and ovaries, which were identified as drawn and described by Kakuda et al. (1995); PECO consisted of the pharynx, esophagus, chelicera, as well as the soft tissues extended from the capitulum, as described in the detailed diagram of muscles connected to capitulum (Gregson, 1960), which included the retractor muscles for the chelicera and the Gené’s organ; ReB refers to the rest of the body including the midgut, salivary glands, rectal sac, Malpighian tubules, tracheae, and the carcass. Tissues from three ticks were pooled for each mRNA extraction.

Tissues were disrupted using the Omni Bead Ruptor-12 Bead Mill Homogenizer (Omni International, Inc., Waterbury, CT, United States) with mixed size (20 of 1.4 mm and 5 of 2.8 mm) ceramic beads for 1 min (ReprT, PECO, and SynG) or 3 min (ReB) at 5.65 m/s at room temperature. Total RNA was extracted from the homogenized tissue using the Zymo Direct-zol™ RNA Microprep Kit (Zymo Research, Irvine, CA, United States) following the protocol of the manufacturer. An extra DNase I treatment was added at the end of the extraction before the final cleanup procedure using a RNA Clean and Concentrator-5 kit (Zymo Research), and total RNA was eluted in 13 μl nuclease-free water. RNA concentration was quantified spectrophotometrically using a Tecan Infinite M200 Pro plate reader (Tecan, Research Triangle Park, NC, United States). For all tissues, cDNA was synthesized using 100–200 ng of total RNA as template, 1 μl oligo(dT)20 (50 mM), and 1 μl random hexamers (50 ng/μl) in 22 μl of final volume reaction using the SuperScript™ III First-Strand Synthesis System (Invitrogen, Carlsbad, CA, United States) following the specifications of the manufacturer.

#### Reverse Transcription Quantitative Real-Time PCR of Pyrokinin Receptor Transcript

The pyrokinin receptor (PKR) nucleotide sequence is highly conserved between *R. sanguineus* and *R. microplus* species. Therefore, reverse transcription quantitative real-time PCR (RT-qPCR) oligonucleotide primers, previously validated for *R. microplus* (Nijhof et al., 2009; Yang et al., 2015; Brock et al., 2019), were used to analyze pyrokinin receptor expression on the tissues mentioned above (Table 1). RT-qPCR was performed using 10 μl reactions, consisting of 5 μl PowerUp SYBR™ Green PCR Master Mix (Applied Biosystems, Inc., Waltham, MA, United States), 1 μl of a primer mix (300 nM of each primer), 2 μl of cDNA (40 ng/μl), and 2 μl of nuclease-free water. All reactions were performed in duplicate. Real-time relative quantification was performed using the QuantStudio™ 6 Pro Real-Time PCR System (Applied Biosystems). The conditions for the RT-qPCR cycles consisted of an initial denaturation step (10 min at 95°C), followed by 40 cycles of 95°C for 15 s and 60°C for 60 s.

**TABLE 1:** Primers used for RT-qPCR.

Primers	Sequence (5′ to 3′)	References	Genes^1^ (GenBank)
RmPKR-qF2	ACGCGCCATGAATGGAA	Yang et al., 2015	*Rhimi-PKR* (KP126932)
RmPKR-qR2	GTGTGAAGCTGGTGGTTTGAGA	Yang et al., 2015	*Rhimi-PKR* (KP126932)
Rhimi-EF1-α-88-F	CGTCTACAAGATTGGTGGCATT	Nijhof et al., 2009	*elf1-*α (EW679365)
Rhimi-EF1-α-196-R	CTCAGTGGTCAGGTTGGCAG	Nijhof et al., 2009	*elf1-*α (EW679365)
RmRPS4-qF1	TCATCCTGCACCGCATCA	Nijhof et al., 2009	*rpl4* (CV436347)
RmRPS4-qR1	ACGCGGCACAGCTTGTACT	Nijhof et al., 2009	*rpl4* (CV436347)

*^1^These genes are from R. microplus and are highly identical the homologous genes in R. sanguineus; therefore, the same primers were used to detect the homologous genes in R. sanguineus with 91–99% efficiency in RT-qPCR.*

Elongation factor 1-alpha (*Rhimi-EF1-*α) and ribosomal protein S4 (*Rhimi-RPS4*) (GenBank accession numbers EW679365.1 and CV436347, respectively) were used as internal reference genes previously validated for the gene expression analysis of *R. microplus* (Nijhof et al., 2009; Yang et al., 2015; Brock et al., 2019). The primer efficiency for amplifying *R. sanguineus* homologous genes was verified using the software LinRegPCR v11 (Academic Medical Center, Amsterdam, Netherlands). The normalized relative quantity (NRQ) was calculated for PKR for each tissue following published formulas (Hellemans et al., 2007). The relative transcript abundance of PKR in each tissue was presented as a fold change (FC) of ReB (FC = 1).

### Statistical Analyses

All statistical analyses and graphs were carried out using the GraphPad Prism 9.0 software (GraphPad Software, La Jolla, CA, United States). Repeated measures one-way ANOVA was used for all contraction assays to compare the tissue responses to different treatments on the same tick (*n* = 5–8 ticks), followed by a Tukey’s multiple comparison test.

For the RT-qPCR statistical analysis, PKR transcript abundance presented as FC of ReB among different tissues (*n* = 3 replicates, each replicate contained tissues from 3 ticks) was determined by the one-way ANOVA test followed by the Tukey’s multiple comparisons test.

## Results

### Annotation of the *capa* Gene From *R. sanguineus*

**FIGURE 1:**
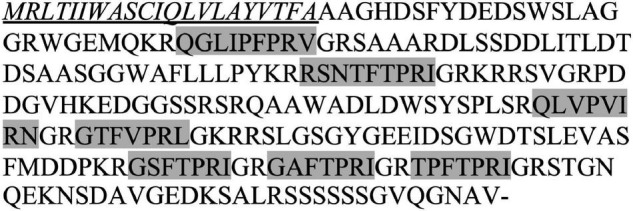
*Rhipicephalus sanguineus* CAPA propeptide. The signal peptide (underlined and italic) was predicted using SignaIP version 5.0, and two periviscerokinins (PVKs) and five pyrokinin (PK) neuropeptides (gray highlights) were predicted by identification of the proteolytic cleavage sites following the rules suggested by Veenstra (2000).

Using the PKR mRNA from the tick *R. microplus* (XM_037421432.1) as the query to perform BLASTn searches against the current available nucleotide collections from *R. sanguineus*, a hit of an uncharacterized protein locus (LOC119383374) was obtained with 100% coverage and 86% sequence similarity. We therefore proposed this locus as *Rhisa*-*capa* gene. The 1,293 bp mRNA (XM_037651475) encodes the 252-amino acid residue CAPA precursor peptide (XP_037507403.1), which has a predicted signal peptide of 21 amino acid residues, two periviscerokinins, and five PKs (Figure 1). The amino acid sequences of these seven neuropeptides encoded by the Rhisa-CAPA precursor are identical to those of *R. microplus*, and Rhisa-CAPA-PVK1, Rhisa-CAPA-PK3, and -PK4 are also identical to those of the tick *Ix. scapularis* (Table 2). In this study, we further annotated a second CAPA-PVK2 in both *R. microplus* and *Ix. scapularis* (Table 2). The slightly derived CAPA-PVK2s of three tick species with RNamide at the C-terminal end are like the first periviscerokinin identified in insects isolated from the American cockroach, *Periplaneta americana* (Pea-PVK-1: GASGLIPVMRNa) (Predel et al., 1995). All three tick species have two PVKs and five PKs encoded by a single *capa* gene, although previously we had reported only one PVK in *R. microplus* and *Ix. scapularis* (Xiong et al., 2021b).

**TABLE 2:** Predicted periviscerokinin and pyrokinins from *Rhipicephalus sanguineus*.

*Rhipicephalus sanguineus*		*Rhipicephalus microplus^1^*		*Ixodes scapularis^1^*	
Rhisa-CAPA-PVK1	pQGLIPFPRVa	Rhimi-CAPA-PVK1	pQGLIPFPRVa	Ixosc-CAPA-PVK1	pQGLIPFPRVa
Rhisa-CAPA-PVK2	pQLVPVIRNa	Rhimi-CAPA-PVK2	pQLVPVIRNa	Ixosc-CAPA-PVK2	MSQQMIPVPRNa
Rhisa-CAPA-PK1	RSNTFTPRIa	Rhimi-CAPA-PK1	RSNTFTPRIa	Ixosc-CAPA-PK1	RSNNFTPRIa
Rhisa-CAPA-PK2	GTFVPRLa	Rhimi-CAPA-PK2	GTFVPRLa	Ixosc-CAPA-PK2	GSFVPRLa
Rhisa-CAPA-PK3	GSFTPRIa	Rhimi-CAPA-PK3	GSFTPRIa	Ixosc-CAPA-PK3	GSFTPRIa
Rhisa-CAPA-PK4	TPFTPRIa	Rhimi-CAPA-PK4	TPFTPRIa	Ixosc-CAPA-PK4	TPFTPRIa
Rhisa-CAPA-PK5	GAFTPRIa	Rhimi-CAPA-PK5	GAFTPRIa	Ixosc-CAPA-PK5	AAFTPRIa

*^1^The sequences of PVK1 and PKs from R. microplus and Ix. scapularis were predicted in a previous publication (Xiong et al., 2021b) and were listed for comparison. CAPA-PVK2s from these two tick species were predicted in this study.*

### Myotropic Activity of Pyrokinins in *R. sanguineus*

**FIGURE 2:**
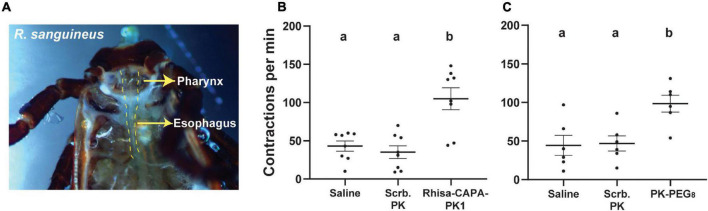
*Rhipicephalus sanguineus* feeding tissue contraction assay. **(A)** Unfed female *R. sanguineus* ticks (2- to 3-month-old) were dissected under tick physiological saline to expose the pharynx-esophagus. Tick endogenous pyrokinin **(B)** and PK analog **(C)** increase the contractions of the pharynx-esophagus: After dissection, ticks were transferred to a Sylgard plate into fresh saline (100 μl). After 5 min of adaptation to the environment, the solution was replaced with 100 μl of saline, and tissues were filmed to register any background contractions. The solution was replaced by 100 μl containing 10 μM of a scrambled peptide of PK (RNFSRINTPa) as negative control and incubated for 3 min before the tissue response was filmed. Tissue was then rinsed (5 × 100 μl of saline), and the saline was replaced with 100 μl of tick endogenous pyrokinin (Rhisa-CAPA-PK1: RSNTFTPRIa), or a PK analog (1944-P8: MS[PEG_8_]-YFTPRLa), both at 10 μM. After 3 min of incubation, the tissue was filmed. Tissue responses to all treatments were filmed for a continuous 1 min using a Lumenera Infinity-1 color camera (Teledyne Lumenera, Ottawa, ON, Canada) installed on an Olympus SZ61 trinocular stereo microscope (Olympus, Center Valley, PA, United States), and contractions in the video were counted by the same operator (mean ± SEM). A repeated measures one-way ANOVA analysis (*P* < 0.05) followed by a Tukey’s multiple comparisons test were performed to determine the difference in response to different treatments on the same tick (*N* = 6–7 ticks); different letters in the figure indicate significant difference, *P* < 0.05.

First, the *in-tissue* activity of Rhisa-CAPA-PK1 (RSNTFTPRIa) and a PK analog (PK-PEG_8_, MS[PEG_8_]-YFTPRLa) was tested at a single high concentration (10 μM). The pharynx-esophagus of *R. sanguineus* (Figure 2A) had a background contraction rate of ∼50 contractions per min (Figures 2B,C); the contraction rate did not change upon stimulation with the scrambled control peptide (RNFSRINTPa). However, the contraction rate increased by a factor of 2 (∼100 contractions per min) in response to endogenous Rhisa-CAPA-PK1 or PK-PEG_8_ tested at 10 μM, significantly higher than responses to the saline and scrambled peptide (*P* < 0.05, Figures 2B,C and Supplementary Video 1). Additionally, we observed the PK and PK analog stimulate the movement of cheliceral digits in *R. sanguineus* ticks (Supplementary Video 2), as an indication for stimulation of the cheliceral muscles.

**FIGURE 3:**
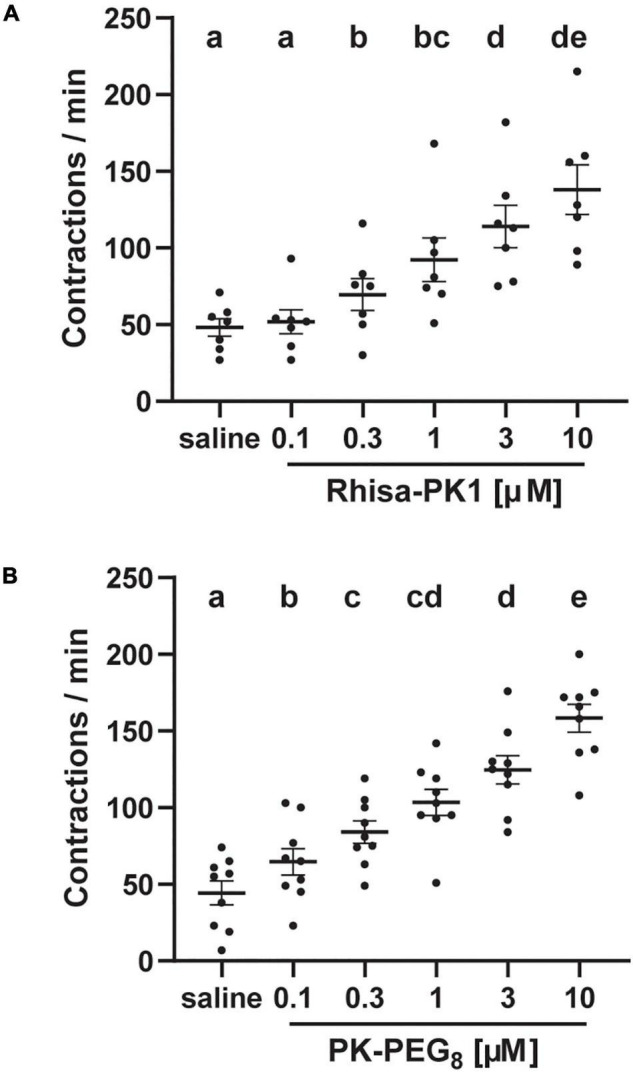
Dose-dependent myotropic activity of a tick endogenous pyrokinin **(A)** and PK analog **(B)** on the pharynx-esophagus of *Rhipicephalus sanguineus*. Unfed female *R. sanguineus* ticks (2- to 3-month-old) were dissected under tick physiological saline to expose the pharynx-esophagus (see Figure 2). After dissection, ticks were transferred to a Sylgard plate into fresh saline (100 μl). After 5 min of adaptation to the environment, the solution was replaced with 100 μl of saline, and tissues were filmed to register any background contractions. Saline was replaced by 100 μl of tick endogenous pyrokinin [Rhisa-CAPA-PK1 (RSNTFTPRIa)], or a PK analog [1944-P8 (MS[PEG_8_]-YFTPRLa)] from low to high concentration. Tissue was rinsed (5 × 100 μl of saline) between different concentrations. Tissue responses to all treatments were filmed for a continuous 1 min at 1 min after the addition of the solution (saline or peptide) using a Lumenera Infinity-1 color camera (Teledyne Lumenera, Ottawa, ON, Canada) installed on an Olympus SZ61 trinocular stereo microscope (Olympus, Center Valley, PA, United States) and contractions in the video were counted by the same operator (mean ± SEM). A repeated measures one-way ANOVA analysis (*P* < 0.05) followed by a Tukey’s multiple comparisons test were performed to determine the difference in response to different treatments on the same tick (*N* = 7–9 ticks), different letters in the figure indicate significant difference, *P* < 0.05.

Furthermore, both Rhisa-CAPA-PK1 and the PK analog elicited dose-dependent stimulatory activity of the pharynx-esophagus tested at five concentrations from 100 nM to 10 μM (Figures 3A,B). Compared with the saline background contractions, Rhisa-CAPA-PK1 increased the pharynx-esophagus contractions starting at 300 nM but not at 100 nM (Figure 3A), while PK-PEG_8_ showed activity at the lowest tested concentration of 100 nM (Figure 3B).

### The Expression of Pyrokinin Receptor in Tissues

**FIGURE 4:**
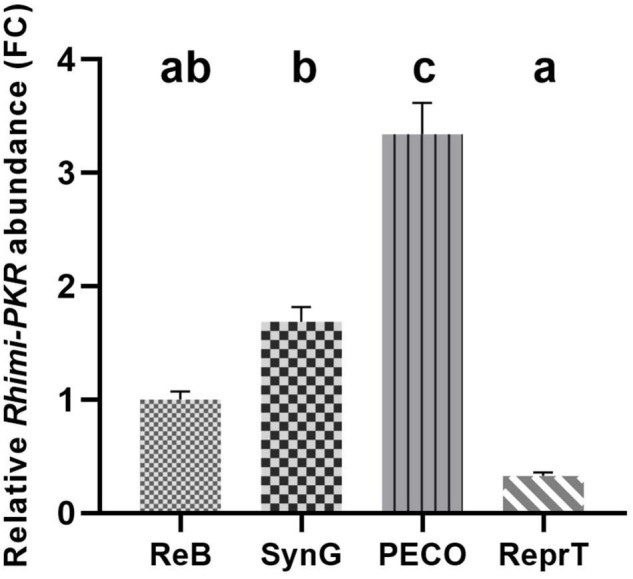
Relative quantification of pyrokinin receptor transcripts in different tissues of *Rhipicephalus sanguineus* using RT-qPCR. The relative abundances of PKR transcripts across different tissues including reproductive tissues (ReprTs), synganglion (SynG), pharynx-esophagus, chelicera, and other soft tissues associated with capitulum (PECO) and the rest of the body (ReB) from unfed adult female of *R. sanguineus* (2–3-month-old) were compared. Tissues from three ticks were pulled for each replicate; a total of 9 ticks were used for three replicates. Two internal reference genes (elongation factor 1-alpha and ribosomal protein S4) were used to normalize the PKR abundance of each replicate. The relative abundance of transcripts in each tissue was expressed as FC of PKR transcript abundance compared with the transcript abundance of ReB (mean ± SEM, *n* = 3). One-way ANOVA followed by a Tukey’s multiple comparisons test was performed to test the difference in relative PKR abundance; different letters in the figure indicate significant difference, *P* < 0.05.

To investigate the relative abundance of PKR in esophagus-pharynx tissue, we analyzed the transcript expression of *R. sanguineus* pyrokinin receptor (XM_037671121) across several tissues of interest using RT-qPCR. These are the feeding-related tissues associated with the capitulum (PECO), ReprTs, SynG, and the ReB. The primers designed based on homologous genes of *R. microplus* (Table 1) showed an efficiency of 91–99% using *R. sanguineus* cDNA as template (data not shown). The level of PKR expression was the highest in the PECO, which included the esophagus-pharynx, chelicerae, and the retractor muscles of the chelicerae; it was 3.3-fold higher than in the ReB (Figure 4). PKR transcripts expression was the lowest in the ReprT in the unfed ticks.

### Myotropic Activity of Pyrokinins in *Ix. scapularis*

**FIGURE 5:**
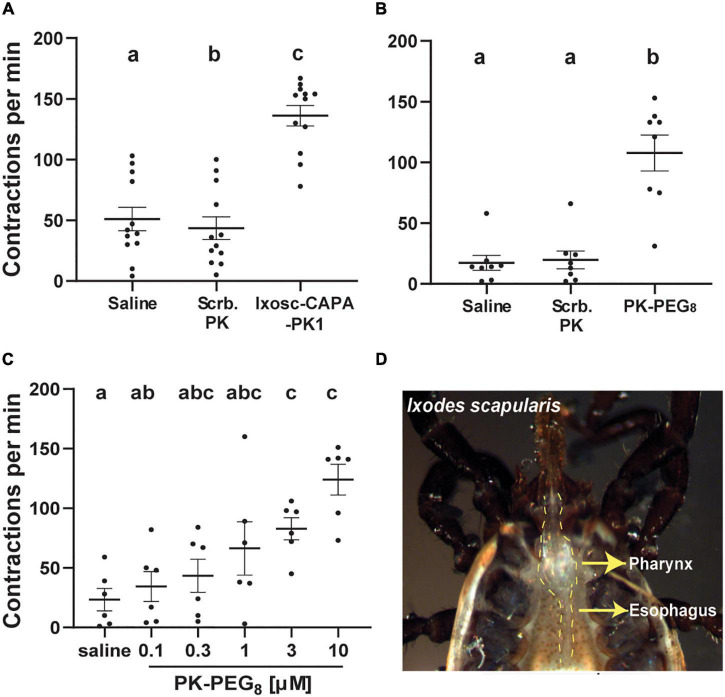
*Ixodes scapularis* feeding tissue contraction assay. **(A)** Activity of the tick endogenous Ixosc-CAPA-PK1 (RSNNFTPRIa) **(B)** and PK-PEG_8_ (MS[PEG_8_]-YFTPRLa). In **(A)**, the PK and in **(B)**, the PK analog increase the contractions of the pharynx-esophagus: After dissection, ticks were transferred to a Sylgard plate into fresh saline (100 μl). After 5 min of adaptation to the environment, the solution was replaced with 100 μl of saline, and tissues were filmed to register any background contractions. The solution was replaced by 100 μl containing 10 μM of a scrambled peptide of PK (RNFSRINTPa) as negative control and incubated for 3 min before the tissue response was filmed. Tissue was then rinsed (5 × 100 μl of saline), and the saline was replaced with 100 μl of Ixosc-CAPA-PK1 or PK-PEG_8_ (both at 10 μM). After 3 min of incubation, the tissue was filmed. **(C)** Dose response of PK-PEG_8_ in the same assay; the tissue was prepared in the same fashion and was incubated for 1 min after addition of each treatment solution and then filmed for the response. Tissue responses to all treatments were filmed for a continuous 1 min using a Lumenera Infinity-1 color camera (Teledyne Lumenera, Ottawa, ON, Canada) installed on an Olympus SZ61 trinocular stereo microscope (Olympus, Center Valley, PA, United States), and contractions in the video were counted by the same operator (mean ± SEM). A repeated measures one-way ANOVA analysis (*P* < 0.05) followed by a Tukey’s multiple comparisons test were performed to determine the difference in response to different treatments on the same tick (*N* = 6–12 ticks); different letters in the figure indicate significant difference, *P* < 0.05. **(D)** Unfed female *Ix. scapularis* ticks (3- to 4-month-old) were dissected under tick physiological saline to expose the pharynx-esophagus.

We further verified the pyrokinin myotropic activity on the same tissues of the blacklegged tick *Ix. scapularis*, another vector tick species. The pharynx-esophagus showed a similar contraction response upon stimulation with the endogenous pyrokinin peptide (Ixosc-CAPA-PK1, 10 μM) or the PK analog PK-PEG_8_ (Figure 5 and Supplementary Video 3). No increase in contractions occurred in response to the scrambled peptide for *Ix. scapularis*, as expected (Figures 5A,B), and the response to the scrambled PK peptide (43 contractions per min) was slightly lower than to the saline (51 contractions per min), *P* = 0.04 (Figure 5A). The tissues unequivocally responded to 10 μM Ixosc-CAPA-PK1 (136 contractions per minute, Figure 5A, *P* < 0.05) and the PK analog (108 contractions per minute, Figure 5B, *P* < 0.05). A dose response in an increased contraction rate to the PK-PEG_8_ from 100 nM to 10 μM was validated on the target tissue (Figure 5C). The tissue started to show a significant increase in the contraction rate when the analog was applied at 3 μM or higher. In addition, we observed movement of the cheliceral digits upon the treatment of PK peptide or the PK analog in ticks whom the cheliceral digits could be seen (Supplementary Video 4).

## Discussion

This is the first study to explore the functional activity of PKs and a PK peptidomimetic in ticks. In this study, we demonstrated the myotropic activity of pyrokinin endogenous ligands as well as a PK analog on two tick vector species, *R. sanguineus* and *Ix. scapularis*. In addition, we annotated the *capa* gene from the brown dog tick, *R. sanguineus*, and quantified the relative PKR transcript abundance in different tissues. The CAPA precursors from *R. microplus* and *Ix. scapularis* were annotated previously (Xiong et al., 2021b). The PVKs and five PK neuropeptides of *R. sanguineus* are identical to that of *R. microplus.* PK3 and PK4 are identical in the three tick species. The tick herein predicted second periviscerokinin has an uncommon ending in RNa. In the DINeR neuropeptide insect database that analyzed 201 species citing 539 PVK corresponding sequences, the ending RNa is only present in the order Blattodea PVKs (e.g., genera *Deropeltis* and *Periplaneta*), with only six such PVKs listed (Yeoh et al., 2017).

The PK and analog appeared to act on the muscles involved in feeding including those of the pharynx-esophagus and the cheliceral muscles, which control the movement of cheliceral digits. Unlike some blood-feeding insects that feed through blood vessels, ticks are pool feeders who evolved a different feeding mechanism. Tick mouthparts (capitulum) comprises the hypostome, chelicera, and the palps. The feeding process of Ixodid ticks involved penetration of the host skin and breaking down of the dermal tissues by the hypostome and retraction of the cheliceral shafts and lateral movement of cheliceral digits (Richter et al., 2013), secreting anti-immune compounds and cementing themselves firmly onto the host (Sonenshine and Roe, 2013), sucking the blood into the food canal and then into the pharynx by contracting the dilator muscles of the pharynx, and when the dilator muscles relax, the blood then pass through the esophagus into the midgut (Vancová et al., 2020). The tick esophagus is a tubular structure connecting the pharynx to the midgut. It passes posterior-dorsally through the SynG and is surrounded by vestiges of the pharyngeal muscles (Gregson, 1960; Šimo et al., 2009). Detailed drawings and the 3D structure of muscles around the pharynx-esophagus structure can be found in the studies by Gregson (1960); Kemp and Tatchell (1971), and Vancová et al. (2020). The epithelial layer of the tick esophagus is surrounded by a muscle layer consisting of both circular and longitudinal muscles (El Shoura, 1988). These muscles could be associated with the peristaltic movement of the esophagus we observed. The pharynx is heavily surrounded by lateral dilator muscles (Vancová et al., 2020). We observed very little background contraction in the pharynx tissue (Supplementary Video 3). It is possible that the increase of pharynx-esophagus contraction was also partially led by the contraction of the dilator muscles of the pharynx. Evidence of pyrokinin signaling system associated with the arthropod feeding tissues was also found in *Drosophila melanogaster*. PKs are encoded by *capability* (*capa*) and *hugin* genes, respectively (Kean et al., 2002; Meng et al., 2002), and *hugin*-expression neurons project axons to the pharyngeal muscles in *Drosophila* larvae (Melcher and Pankratz, 2005; Schoofs et al., 2014). Hugin PK is necessary for regulating feeding in *Drosophila* larvae (Schlegel et al., 2016).

In addition to the pharynx-esophagus, we observed that the PK and the PK analog stimulated the movement of the cheliceral digits in both *R. sanguineus* and *Ix. scapularis* (Supplementary Videos 2, 4); this indicated that the PKs acted on the chelicera-related muscles (Gregson, 1960; Kemp and Tatchell, 1971). It is noted that the SynG was removed from the tick during dissection, so, the increased contraction of the target tissues was not likely due to neuronal stimulation. In summary, the myotropic activity of PKs on the tissues associated with blood meal uptake appeared to be conserved in the two hard tick lineages, namely, Prostriata (*Ix. scapularis*) and Metastriata (*R. sanguineus*) (Anderson et al., 2004).

The PK analogs with enhanced bioavailability and biostability are promising tools for novel pest control. From our previous study in which endogenous PK ligands and twenty PK analogs were tested on the recombinant *R. microplus* PK receptor, the analog PK-PEG_8_ (EC_50_ = 401 nM) used in this work had been the most potent of the tested analogs but was still 4 times less potent than the Rhimi-CAPA-PK1 (same sequence as Rhisa-CAPA-PK1 tested in this study, EC_50_ = 101 nM) (Xiong et al., 2021b). In contrast, in the *in-tissue* assay performed herein, the pharynx-esophagus responded to PK-PEG_8_ at lower concentration (100 nM) than to the Rhisa-CAPA-PK1, which responses started at 300 nM. This can be explained by the polyethylene glycol (PEG) polymer conjugated to the N-terminus, which confers enhanced penetration across cell membranes (Boccu et al., 1982; Shen et al., 2009) and improves the digestive enzyme resistance of bioactive peptides (Jeffers and Roe, 2008). A PEG-analog of PK antagonist (PK-dF-PEG_8_: MS[PEG_8_]-YF[dF]PRLa) induces antifeedant activity in the pea aphid (Nachman et al., 2012). We previously identified other analog candidates with tick receptor agonistic activity, which will be tested in the future (Xiong et al., 2021b).

In accordance with the found functional activity of PK peptide and analog on the pharynx-esophagus and cheliceral digits, the PKR transcripts were most abundant in these feeding-related tissues. The second tissue in which the receptor was most abundant was the SynG. In the unfed *Ix. scapularis* adult females, Gondalia et al. (2016) showed that among all tissues analyzed, the PKR is most abundant in the SynG, but they did not specifically investigate the pharynx-esophagus or cheliceral muscles. The tick mouthparts are an attractive target for interfering with tick feeding and therefore blocking pathogen transmission. Previously, research on tick GPCRs has been mostly focused on the GPCR ligands acting on the salivary glands to regulate salivary gland contraction and saliva secretion (Šimo et al., 2012, 2014; Vancová et al., 2019). In this study, we reported a new target site for potential feeding disruption by identifying the pyrokinin activity in the muscles associated with the pharynx-esophagus, lateral dilator pharynx muscles, and most likely, the cheliceral muscles. The target sites of the PK in these tissues should be further investigated by immunolocalization.

This study represents the first report on tick pyrokinin function. We developed a model myotropic *in-tissue* tick assay to validate the functional activity of PK ligands. As pool feeders, ticks must acquire blood through the preoral canal and into the pharynx by rhythmic contraction of muscles of feeding tissues (Vancová et al., 2020). We thus proposed that pyrokinins regulate, at least partially, the pharynx and esophagus rhythmic contractions that are critical for tick blood ingestion. Therefore, the pyrokinin signaling system appears as a potential target for tick feeding interference to prevent pathogen acquisition.

## Data Availability Statement

The original contributions presented in the study are included in the article/Supplementary Material, further inquiries can be directed to the corresponding author/s.

## Author Contributions

CX, JW, and PP conceptualized the experiments and wrote and edited the manuscript. CX and JW performed the experiments and statistical analyses. RN designed and synthesized the hyperpotent analog, provided comments/suggestions on the text, and edited the manuscript. All authors contributed to the article and approved the submitted version.

## Conflict of Interest

The authors declare that the research was conducted in the absence of any commercial or financial relationships that could be construed as a potential conflict of interest.

## Publisher’s Note

All claims expressed in this article are solely those of the authors and do not necessarily represent those of their affiliated organizations, or those of the publisher, the editors and the reviewers. Any product that may be evaluated in this article, or claim that may be made by its manufacturer, is not guaranteed or endorsed by the publisher.
