# Supplementary material for: Myotropic Activities of Tick Pyrokinin Neuropeptides and Analog in Feeding Tissues of Hard Ticks (Ixodidae)
Source: Front Physiol. 2022 Feb 15;12:826399. doi: 10.3389/fphys.2021.826399 (PMC8887807; doi:10.3389/fphys.2021.826399)
Supplement: Supplementary file 1 [file Table_1.DOCX]

**Supplementary information**

Supplementary_video_01: Contractions of *R. sanguineus* pharynx-esophagus in response to PK-PEG_8_ at 10µM (Tick5 from 10/06/21)

Supplementary_video_02: Movement of *R. sanguineus* cheliceral digits in response to Rhisa-CAPA-PK1 at 10µM (Tick6 from 08/31/21)

Supplementary_video_03: Contractions of *Ix. scapularis* pharynx-esophagus in response to PK-PEG_8_ at 10µM (Tick2 from10/05/21)

Supplementary_video_04-v2: Movement of *Ix. scapularis* cheliceral digits in response to PK-PEG8 at 10µM (Tick2 from 06/22/21)
